# Dental management of pediatric patients affected by pulmonary atresia with ventricular septal defect: A scoping review 

**DOI:** 10.4317/medoral.21864

**Published:** 2017-06-18

**Authors:** Arturo Garrocho-Rangel, Ana-Cristina Echavarría-García, Miguel-Ángel Rosales-Bérber, Joselín Flores-Velázquez, Amaury Pozos-Guillén

**Affiliations:** 1 DDS, MSc, PhD, Especialidad en Estomatología Pediátrica, Facultad de Estomatología, Universidad Autónoma de San Luis Potosí, San Luis Potosí, S.L.P., México; 2 DDS, Especialidad en Estomatología Pediátrica, Facultad de Estomatología, Universidad Autónoma de San Luis Potosí, San Luis Potosí, S.L.P., México; 3 DDS, MSc, Especialidad en Estomatología Pediátrica, Facultad de Estomatología, Universidad Autónoma de San Luis Potosí, San Luis Potosí, S.L.P., México; 4 DDS, Especialidad en Estomatología Pediátrica, Facultad de Estomatología, Universidad Autónoma de San Luis Potosí, San Luis Potosí, S.L.P., México; 5 DDS, MSc, PhD, Laboratorio de Ciencias Básicas, Facultad de Estomatología, Universidad Autónoma de San Luis Potosí, San Luis Potosí, S.L.P., México

## Abstract

**Background:**

Congenital Heart Diseases (CHD) involves a wide range of pathological conditions, such as Pulmonary Atresia with Ventricle Septal Defect (PA/VSD). This disorder leads to the systemic circulation of oxygen-poor blood (cyanosis), with associated features and consequences in the oral cavity.

**Material and Methods:**

Using scoping review methodology for screening and article selection, the primary objectives of this paper were as follows: first, to pose a research question; second, to identify relevant studies in order to answer the research question; third, to select and retrieve the studies; fourth, to chart the critical data, and finally, to collate, summarize, and report the results from the most important articles on the dental management of children affected with PA/VSD. Relevant articles (Randomized Controlled Trials [RCT], reviews, observational studies, and clinical case reports) published over a 10-year period were identified and retrieved from four Internet databases: PubMed; Embase/Ovid; Cochrane Library, and Google Scholar.

**Results:**

By title and abstract screening and after removing duplicates, 24 articles were finally included in the present scoping review. According to the extracted data, the following are the most important clinical issues to be considered when treating children with PA/VSD in the dental setting: prevalence of dental caries; prevention of dental disease (oral hygiene and diet); bacteremia and infective endocarditis risk, and child behavior control and treatment under general anesthesia.

**Conclusions:**

Pediatric Dentists should bear in mind that early diagnosis and treatment, together a long-term follow-up of children with PA/VSD, continue to be the best approaches for achieving enhanced patient psychological well-being and, in consequence, their good quality of life.

** Key words:**Pulmonary atresia, congenital heart diseases, ventricle septal defect, scoping review, dental management.

## Introduction

Tetralogy of Fallot (TF) is the most common form of Congenital Heart Disease (CHD), which is characterized by the association of four cardiac abnormalities (1,2): (i) maligned ventricular septal defect; (ii) sub-pulmonary stenosis; (iii) overriding aorta, and (iv) right ventricular hypertrophy. TF accounts for around 10% of all CHD, with a prevalence of >2 per 10,000 births, with males affected more than females (3-5).

Pulmonary Stenosis with Ventricular Septal Defect (PS/VSD) in pediatric patients represents the most severe form of this disease spectrum (1). In these cases, no direct communication exists between the heart right ventricle and the lungs; consequently, there is a partial or complete obstruction of the blood flow from right ventricle to the pulmonary circulation. Thus, to maintain blood flow to the lung, higher ventricle pressure is generated, which is manifested as compensatory right ventricle hypertrophy. These abnormal events lead to the systemic circulation of oxygen-poor (desaturated) blood (6). In severe cases, a right-to-left shunting through the foramen ovale is formed, which is manifested by cyanosis, acropachy, polycythemia, systemic hypoxia, anemia, bluish appearance, and clubbing of the fingers (4,6). The etiology of TF continues to remain unclear, but it has been related with defects occurring from week 3 to week 8of intrauterine life (7,8). Likewise, several associated conditions have been mentioned as follows: low birth weight; short gestational duration; rubella; maternal infections; smoking or alcoholism; drugs such as Thalido-mide, Warfarin, and Phenytoin, and, possibly, twinning (4,8). Gene mutations have been suggested in 4% of TF cases (9). Approximately 21% of TF have been associated with diverse syndromes, for instance, Down syndrome, Noonan syndrome, Branchial Arch syndrome, and chromosome 22q11 microdeletion (4,10). During the last 20 years, medical and surgical techniques have substantially improved, and >80% of affected children reach adulthood (10); pediatric survival is rare in the absence of surgical intervention and depends mainly on the adequacy of the patient’s pulmonary collateral circulation.

Similar to other congenital heart defects, PSD/VSD impacts upon dental health with respect to three different issues: dentition development; risk of infective endocarditis from bacteremia induced by invasive dental procedures, and special implications with treatment delivered when required (1,3,11). Regarding the first point, ameloblasts are extremely sensitive to metabolic alterations, for example, in cases of CHD, during tooth formation, which can lead to the formation of a thinner and/or softer enamel tissue; in consequence, these teeth are more susceptible to faster destruction due to caries and are more difficult to restore. Other oral abnormal findings include delayed tooth eruption, stomatitis, glossitis, cyanotic mucus membrane, tongue, and gingiva (7,12). Therefore, it is necessary to conduct an early oral examination, followed by the promotion of individual hygiene measures and the implementation of therapeutic procedures in children affected by PSD/VSD (11,13). Despite recent medical progress, CHD are still a cause of high mortality in pre-school children, accounting for one half of deaths in this age group (8). In this context, the present article aims to present the main results of a scoping review on the dental management of patients with PSD/VSD performed over a period of the last 10 years.

## Material and Methods

A scoping review was conducted between November 2016 and January 2017. We implemented the Arksey and O’Malley methodological framework and recommendations for this type of review (14).

- Research question

The present review intended to answer the following research question: What are the current best dental management approaches for children and adolescents suffering from pulmonary atresia with ventricular septal defect? 

- Identifying relevant studies

The present scoping review’s primary objectives were the following: to pose a research question; to identify and retrieve relevant studies; to select studies relevant to the research question in order to chart critical data from the selected studies, and finally, to collate, summarize, and report the results from the most important articles on PA/VSD in children and adolescents that were published between January 1990 and December 2016, in order to answer the previously posed research question. These articles were accessible on four Internet electronic databases: MEDLINE (via PubMed); Embase/Ovid; the Cochrane Library, and Google Scholar. The search strategy was appropriately adapted for each database. The main search terms, MeSH, or free-text terms, keywords, and Boolean operators, alone or in combination, chosen for these aims included “congenital heart disease”, “tetralogy of Fallot”, “pulmonary atresia”, “ventricular septal defect”, “cyanosis”, “children”, “adolescents”, “pediatric dentistry”, “dentistry for children”, and “pedodontics”. The filter ‘Age’ was set at ‘Child: birth-18 years’. The search algorithm is described in  Table 1.

**Table 1:**
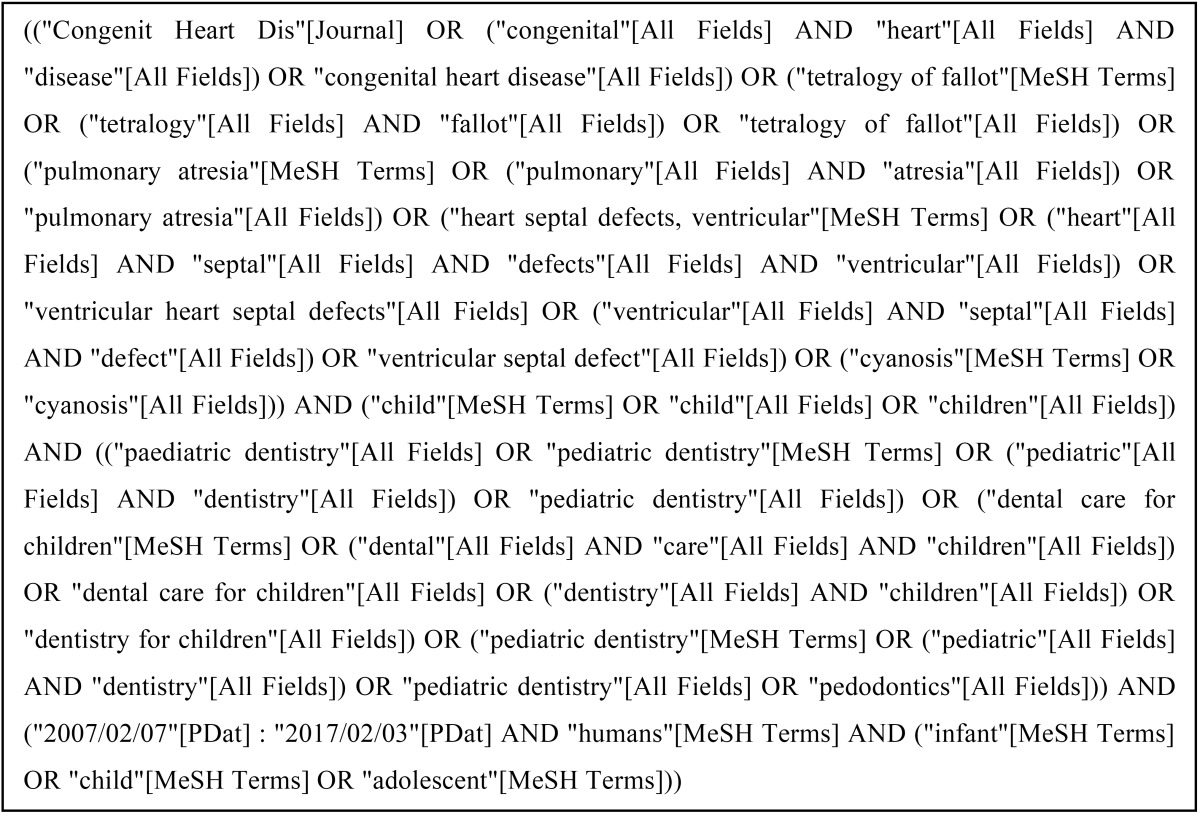
Search strategy.

- Study screening and selection

**Figure 1:**
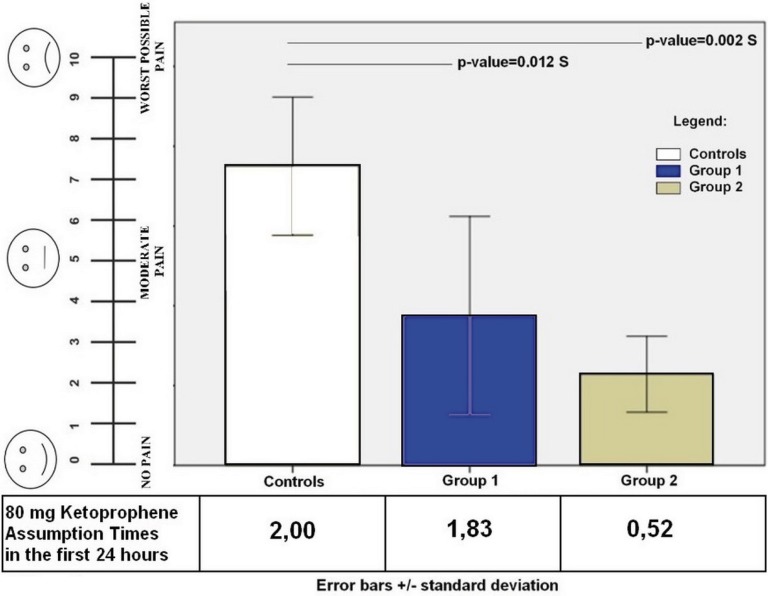
Flow diagram of eligible studies.

The titles and abstracts that were considered reliable, derived from electronic searches, were carefully reviewed by three authors (ACE-G, JF-V, and MAR-B), by independent screening, to select the most relevant studies for inclusion in the review. Different types of peer-reviewed publications, in the English or Spanish languages, were screened as follows: prospective clinical trials (parallel groups or cross-over designs); observational or descriptive studies (cohort, case-control, and cross-sectional studies); pilot studies; narrative reviews of the literature; *in vitro* studies; clinical case series, and case reports. The following articles were excluded: studies involving solely adults; patients with other systemic/syndromic conditions, and purely laboratory investigations. A complementary hand-search in the reference lists of selected articles was also performed; then, the eligible articles, in their full-text form, were obtained. The reported outcome extraction process –employing a predesigned and standardized form– was carried out by other two experienced authors (JAG-R and AJP-G), also in an independent manner, and any disagreement or discrepancy was resolved by discussion and consensus. Specifically, relevant clinical information on the pediatric dental management of PA/VSD was searched for extraction, such as diagnostic methods, oral hygiene/prevention approaches, dental consequences of the disease, special medical considerations (including potential side effects), restorative/pulpal/surgical interventions in primary and young permanent teeth, orthodontics/dentofacial-orthopedics treatment modalities, and oral health maintenance (control and follow-up). A judgment concerning whether each outcome was primarily clinician-centered was also carried out. The flow chart for the entire article-search process is depicted in Figure 1. Finally, a descriptive statistical analysis for the studies included was performed. This analysis involved relevant methodological characteristics from each chosen study, such as design, aim, sample size, population type, follow-up period, and others; also, main clinical findings, medical considerations and dental management were included. These features were summarized through specific  Table 2, Table 2 continue- Table 4.

**Table 2:**
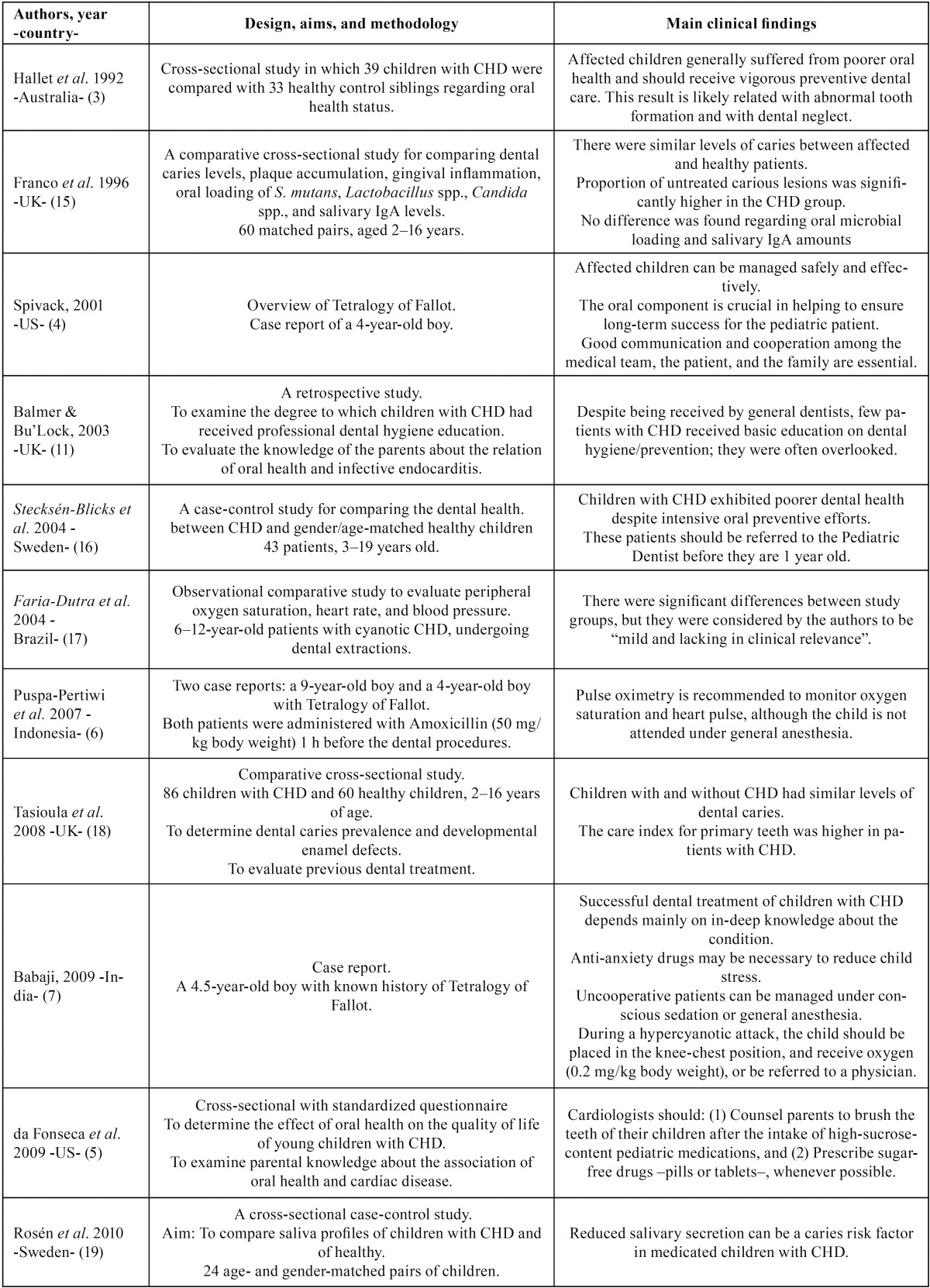
Characteristics and main findings reported from selected studies.

**Table 2 continue:**
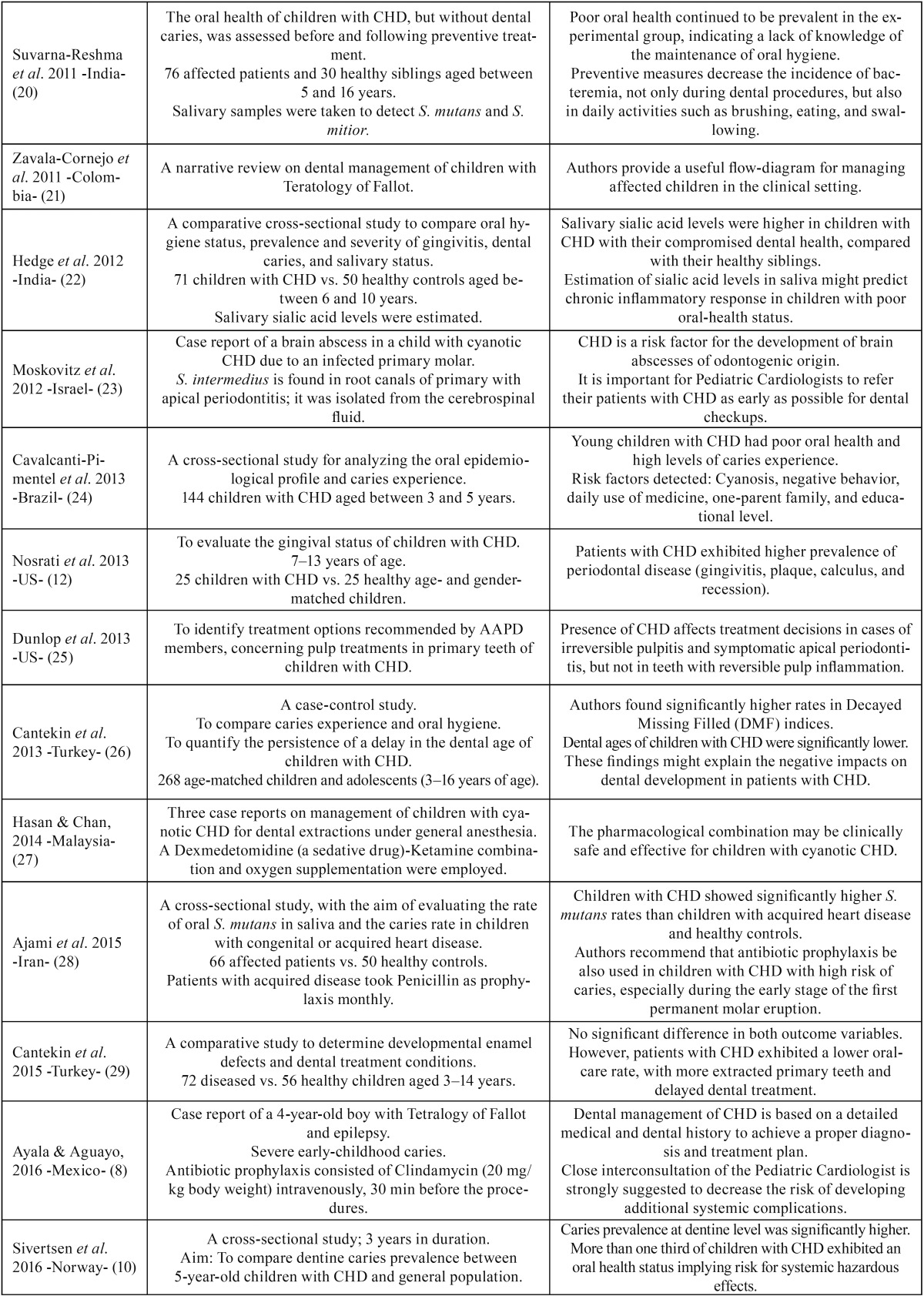
Characteristics and main findings reported from selected studies.

**Table 3:**
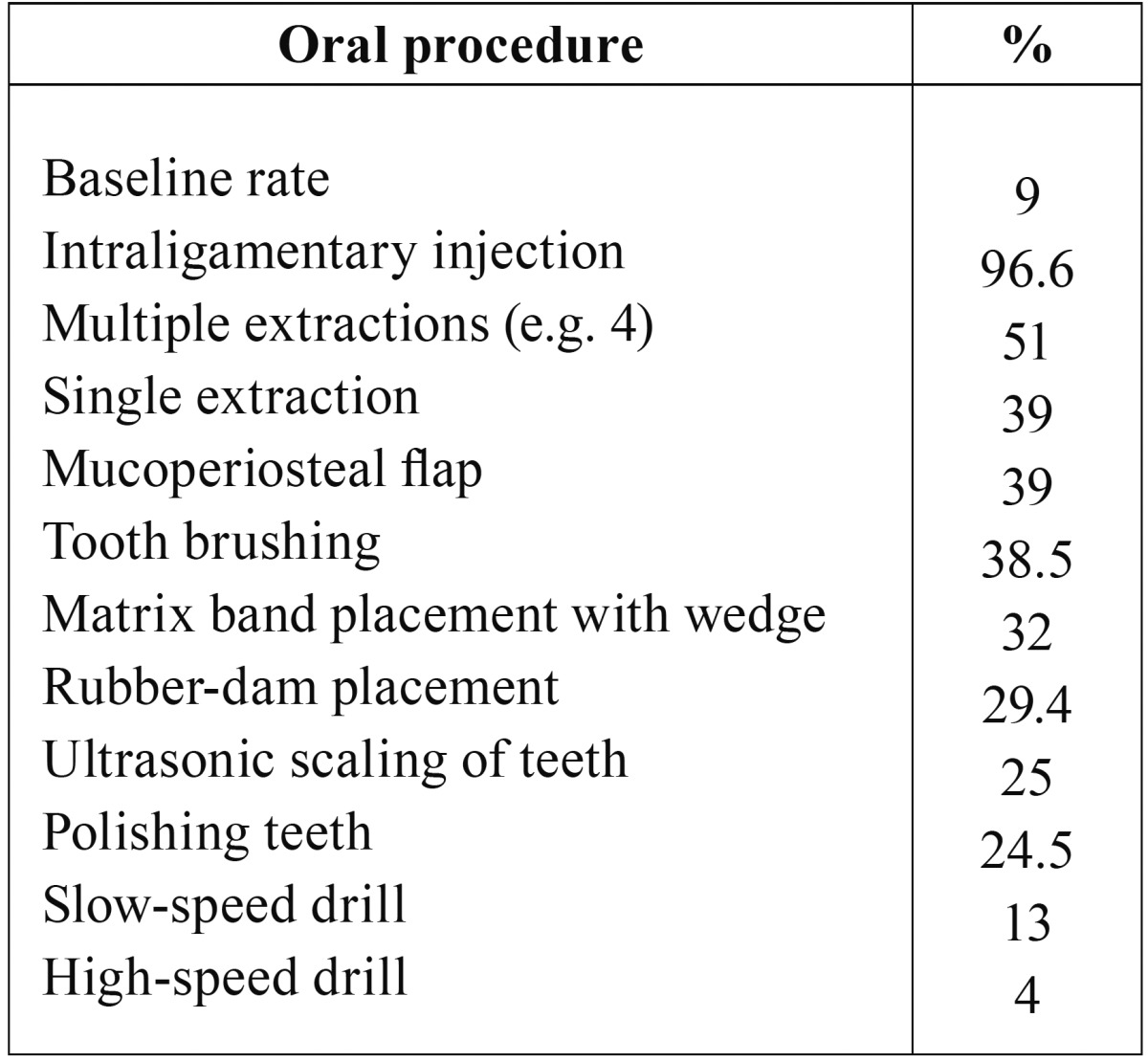
Incidence of bacteremia following diverse dental procedures on primary teeth (35,37).

**Table 4:**
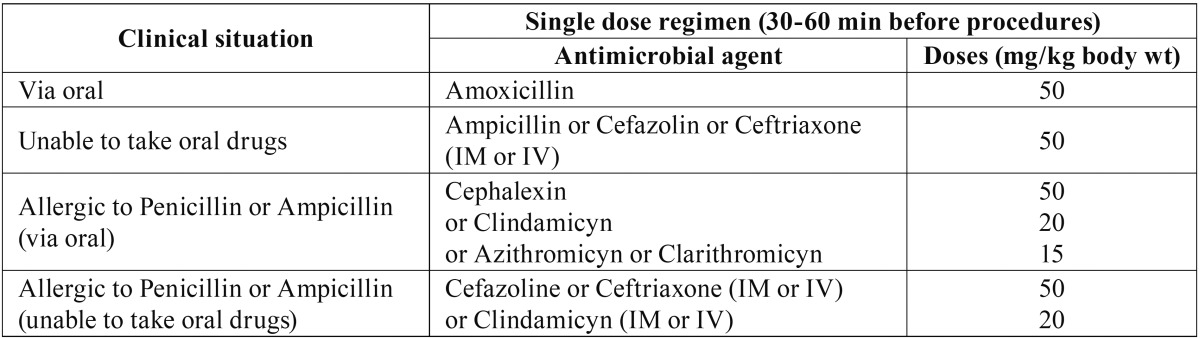
Antibiotic prophylaxis regimens for dental procedures in children -adapted from AAPD (13) and Zavala-Cornejo *et al.* (21).

## Results

- Charting the Data

The initial electronic database search yielded a total of 96 potential articles. By title and abstract screening and after removing duplicates, 41 articles were included. These were retrieved in full-text and analyzed; an additional hand-search was also per-formed. Finally, 24 articles were included in the present scoping review (23 in English and 1 in Spanish) for the pertinent information-extraction process. Figure 1 describes the flow diagram of eligible studies.

- Collating, Summarizing, and Reporting the Results

After exploring the final selection of articles, a large amount of relevant clinical information was able to be condensed. Main findings from this process are outlined as follows (see  Table 2) (15-29).

## Discussion

Knowledge synthesis is currently essential to advance clinical health-science practice and research through consolidation of evidence and to aid practitioners to make a more efficient process of evidence-base decision (30,31). In this regard, scoping reviews are increasingly undertaken as a popular approach to review sufficient health-research evidence (14,32). This type of literature review involves the synthesis and analysis of a wide range of literature and research, including clinical oral health care (31), with rapid ‘mapping’ of the key concept from the relevant literature and providing general conceptual clarity about a specific topic or field of evidence (14,33). Scoping reviews are different from and more flexible in terms of design than systematic reviews. First, systematic reviews focus on a well-defined question and appropriate specific study designs can be identified in advance, while scoping reviews tend to address broader topics in which different study designs may be applicable. In second place, systematic reviews aim to provide answers to the posed question from quality assessed studies; a scoping review does not intend to address very specific questions, nor, consequently, it does not incorporate a quality assessment of the selected primary studies (31). The significance of scoping reviews lies in their main purposes as follows: (i) to examine the extent, range, and nature of research activity; (ii) to determine the value of performing a systematic review; (iii) to summarize and disseminate relevant research findings, and (iv) to identify research gaps in the existing health literature (14,30,32,33).

According to the results of the present review, the following four main clinical considerations may be established and discussed in order to increase the probability of achieving proper management of CHD-affected children in clinical pediatric dentistry, specifically those with pulmonary atresia with ventricular septal defect: (i) prevalence of dental caries; (ii) prevention of dental disease (oral hygiene and diet); (iii) bacteremia and infective endocarditis risk, and (iv) child behavior control and treatment under general anesthesia.

(i) Prevalence of dental caries. Studies on dental caries prevalence in children with PA/VSD have shown variable results (11). Hallet *et al.* (3) reported increased higher levels of caries in affected children when compared with healthy siblings. Similar results were reported in two studies performed with pre-school participants in Sweden (16) and Brazil (24). Also, significantly higher levels of salivary *Streptococcus mutans* levels and caries rates were detected in Iranian affected patients (28). However, Franco *et al.* (15) found no differences in decayed/missing/filled teeth (dmft) or Decayed/Missing/Filled Teeth (DMFT) between cardiac and control children aged 2–16 years. Ayala & Aguayo (8) mention that among patients with PA/VSD, those treated with Digoxin demonstrate a greater number of carious lesions. On the other hand, a consistent outcome found in the present scoping review was the higher degree of untreated caries, delayed treatment, and decreased level of dental care in patients with PA/VSD (1,11,15,26,29). These conditions may lead to multiple extractions of primary teeth, possibly requiring general anesthesia (24,26).

(ii) Prevention of dental disease (oral hygiene and diet). Maintaining oral health in children with PA/VSD is extremely important because the oral cavity is the chief entrance via for bacteria that may cause infective endocarditis, which increases the risk of additional damage to the heart condition (1). Chronic administration of liquid sucrose-sweetened oral drugs increases the incidence of dental caries and gingivitis, particularly in young children and in cases of developmental enamel defects (8,20). Additionally, administered diuretics may lead to impaired salivary secretion or xerostomia (16,19). Further, many children with PA/VSD have difficulties with feeding and nutrition, particularly during their first year of life, mainly vomiting during breastfeeding, reduced appetite, and low weight gain due to increased energy consumption (10). In order to compensate for these deficiencies, parents usually pamper their children with frequent meals based on glucose/fat foods or drinks; night meals are often necessary to maintain an acceptable level of energy intake (10,16,22).

Thus, an aggressive preventive/restorative program must be implemented in these patients as early as possible, focused primarily on exhaustive oral hygiene, fluoride varnish applications, dietary counseling, pit and fissure sealants, maintenance of good gingival/periodontal health, and the eradication of carious lesions and local infection processes (6,11-13,16).

(iii) Bacteremia and infective endocarditis risk. Approximately one-eighth of children with CHD may develop infective endocarditis if exposed to oral bacteremia after a dental procedure (1,6,11). The most common bacteria, associated in >60% of patients with endocarditis and found in the oral cavity and pulp canals of infected primary teeth, are *Staphylococcus aureus*, group B (Viridans) streptococci (e.g., *Streptococcus mutans, Streptococcus sanguis*, and *Streptococcus mitis*), and enterococci (20,23,34). These bacteria may reach the bloodstream through the dental pulp and periodontal pockets (10). Patients with a history of repaired or unrepaired CHD, rheumatic heart fever, transplants, or presence of artificial heart valves, implants, shunts, conduits, or catheters may require antibiotic prophylaxis prior to any invasive dental treatment (e.g., deep scaling, extractions, pulpotomy/pulpectomy, clamping and banding, or any procedure involving manipulation of gingival or periapical tissues or perforation of oral mucosa) ( Table 3) (6,13,18,34,35-37).

According to Hallet et al. (3), there is an increased risk of endocarditis from residual infection in primary tooth canals, which are difficult to debride thoroughly. That is the reason that several authors suggest the extraction/space maintainer vs. a pulpectomy procedure in primary teeth; they recommend endodontic procedures only in permanent teeth with straight canals and closed apex (7). According to Jain *et al.* (36), incidence and magnitude due to canal instrumentation not extending to the periapical tissue are very low, and nearly all bacteria are eliminated from the blood within 10 min. Poor oral hygiene affects the frequency of oral bacteremia following daily practices such as chewing, flossing, and brushing. Different antibiotic prophylaxis regimens for dental procedures in children are depicted in  Table 4 (13,21).

(iv) Child behavior control and treatment under general anesthesia. Severely affected children may have reduced tolerance to the stress or anxiety when dental treatment is provided. Thus, dental treatment should be administered under stress-free conditions, thereby preventing cyanotic events (4,8). Very poorly cooperative patients may be managed with either conscious sedation or under general anesthesia (7). This latter procedure poses many challenges to Anesthesiologists because of the diverse problems associated with CHD (e.g., chronic hypoxemia, risk of brain abscess, pulmonary hypertension, and bacterial endocarditis), mainly due to the anesthetic technique and to perioperative drugs, which can affect the patient’s physiologic status during surgery (27). Also, general anesthesia may require additional financial resources (e.g., especial care and hospitalization), together with psychological and physical burdens for the child and her/his family (11).

Strengths and weaknesses. The current scoping review carefully followed the recommendations stated by Arksey and O’Malley (14) for screening papers and extracting relevant data from them. However, as in any scoping review, a significant weakness was that a critical methodological quality and the risk of bias of each article could not be assessed. Additionally, only English and Spanish language articles were reviewed; despite the latter limitation and the small number of published articles regarding pulmonary atresia with ventricular septal defect in the pediatric dentistry field, we are confident that sufficient reliable and useful information could be collected and synthesized, with the purpose of aiding clinicians to better understand this pathological condition.

- Additional clinical recommendations (1,3,4,7,20,21):

• A thorough review is essential of the child’s history, symptoms, and current medical status.

• Children should be instructed to avoid vigorous brushing.

• Ideally, dental treatments should be provided during the morning, during short appointments, with 2–4 week intervals between these.

• Before cardiac surgery, the child is dentally treated to eliminate potential or active oral infective sites.

• Patient’s oral cavity should be rinsed with 0.2 chlorhexidine gluconate before any dental procedure.

• If a hypercyanotic attack occurs, the child should be placed in a knee-chest position, administer oxygen (0.2 mg/kg body weight), and refer to the Pediatrician.

• Practitioners must take in account potential drug interactions with chronic medications administered to affected patients, particularly in those with severe hematological (coagulation), respiratory, and immunological problems.

## Conclusions

The relationship between oral and systemic health should be consistently reinforced, especially to parents of children with cardiac diseases. Thus, Pediatric Dentists, Cardiologists, and other associated health practitioners should work together in order to educate children affected with PA/VSD and their parents and also to enhance oral health-related quality of life in this vulnerable popula-tion.
